# *Ex vivo* expansion in a clinical grade medium, containing growth factors from human platelets, enhances migration capacity of adipose stem cells

**DOI:** 10.3389/fimmu.2024.1404228

**Published:** 2024-05-14

**Authors:** Francesco Agostini, Carla Vicinanza, Elisabetta Lombardi, Francesco Da Ros, Miriam Marangon, Samuele Massarut, Mario Mazzucato, Cristina Durante

**Affiliations:** ^1^ Stem Cell Unit, CRO Aviano, National Cancer Institute, IRCCS, Aviano, Italy; ^2^ Breast Cancer Surgery Unit, CRO Aviano, National Cancer Institute, IRCCS, Aviano, Italy

**Keywords:** adipose tissue, mesenchymal stem/stromal cells, migration, chemiotaxis, millifluidic transwell assay, drug delivery, good manufacturing practice, supernatant rich in growth factors

## Abstract

**Introduction:**

Adipose tissue mesenchymal stem/stromal cells (ASC) can be used as advanced therapy medicinal product in regenerative and cancer medicine. We previously demonstrated Supernatant Rich in Growth Factors (SRGF) can replace fetal bovine serum (FBS) to expand ASC by a clinical grade compliant protocol. The therapeutic potential of ASC is based also on their homing capacity toward inflammatory/cancer sites: oriented cell migration is a fundamental process in this scenario. We investigated the impact of SRGF on ASC migration properties.

**Methods:**

The motility/migration potential of ASC expanded in 5% SRGF was analyzed, in comparison to 10% FBS, by standard wound healing, bidimensional chemotaxis and transwell assays, and by millifluidic transwell tests. Mechanisms involved in the migration process were investigated by transient protein overexpression.

**Results:**

In comparison to standard 10% FBS, supplementation of the cell culture medium with 5% SRGF, strongly increased migration properties of ASC along the chemotactic gradient and toward cancer cell derived soluble factors, both in static and millifluidic conditions. We showed that, independently from applied migratory stimulus, SRGF expanded ASC were characterized by far lower expression of α-smooth muscle actin (αSMA), a protein involved in the cell migration machinery. Overexpression of αSMA induced a significant and marked decrease in migration capacity of SRGF expanded ASC.

**Discussion:**

In conclusion, 5% SRGF addition in the cell culture medium increases the migration potential of ASC, reasonably through appropriate downregulation of αSMA. Thus, SRGF could potentially improve the therapeutic impact of ASC, both as modulators of the immune microenviroment or as targeted drug delivery vehicles in oncology.

## 1 Introduction

Mesenchymal stem/stromal cells (MSC) are characterized by the ability to sustain cell growth and homeostasis and by the property to balance inflammatory conditions in the microenvironment (1). Moreover, such cells can reach primary and metastatic cancer masses after intravenous infusions (2). In virtue of such homing properties, MSC can be exploited as a potential drug delivery vehicle for therapeutic purposes in cancer patients. Fabrication of an advanced therapeutic medical product (ATMP), based on MSC, requires an efficient cell expansion protocol, in compliance with good manufacturing practice (GMP) guidelines. As previously published (3), we described a GMP compatible protocol to efficiently expand adipose tissue derived MSC (ASC). The protocol was based on the introduction, in the cell culture medium, of a medium additive, that we defined as Supernatant Rich in Growth Factors (SRGF). Sterile SRGF was derived from human platelet concentrates, in which growth factors were released from thrombocytes by the addition of CaCl_2_, as platelet activation trigger (4). Reliability and consistency of SRGF biologic functions were demonstrated in a previous work (5), in which we showed that such medium additive can be fully standardized pooling together at least 16 products derived from single donors. For such reasons, SRGF can be considered as compliant with GMP guidelines for ATMP fabrication. SRGF was shown to be more efficient than other similar medium additives, as e.g. platletet lysate, in promoting growth of MSC (6). ASC expanded in presence of SRGF were also shown to strongly interact, in microfluidic conditions, with selected types of cancer cells, suggesting a peculiar affinity of such ASC for target tumors (7). Nevertheless, the MSC homing process from circulating blood to cancer cells is not limited to direct cell-cell interaction but it’s also characterized by oriented migration through a chemotactic gradient toward the cancer target (2). During cell migration, cells extend protrusions, shaped by actin, that adhere to the extracellular matrix through integrin clusters, defined as focal adhesions (8). Focal adhesions are highly multifaceted protein complexes in which few factors can play pivotal roles. Paxillin (PAX) is considered as a primary scaffold in the architecture of the focal adhesion protein complex (9). PAX was previuosly shown to be recruited at adhesion sites located in the forward part of a moving cell (10). Vinculin (VINC) and PAX provide a connection between actin and integrins: in particular, VINC is a mechano-sensing protein and its downregulation was linked to increased cell motility in bidimensional conditions (11). Both VINC and PAX play a crucial role in modulating the interaction with Focal Adhesion Kinase (FAK) (12, 13). FAK is a phosphorylation activated enzyme controlling formation and disassembly of focal adhesion (14). Previous publications showed α-smooth muscle actin (αSMA) is required for cell diapedesis (15), even though it was also shown to inhibit migration of selected primary cells (16). We previously showed (17) that FilaminA (FLNA) is increased in fast migrating cells, as MSC derived from the bone marrow of multiple myeloma patients. In this work, we aimed to investigate the impact of SRGF on the migration potential of expanded ASC. Thereafter, we also attempted to investigate molecular mechanisms explaining SRGF mediated effect on the migratory potential of expanded ASC.

## 2 Methods

### 2.1 Cell isolation and expansion

ASC were purified from stromal vascular fraction isolated from lipoaspirates obtained from female breast cancer patients that underwent mammary reconstruction by lipofilling after mastectomy. The study was in accordance with the Declaration of Helsinki (2004) and it was approved by Ethics Committee of CRO Aviano, National Cancer Institute, IRCCS (protocol number: CRO-2016–30). The study was conducted in accordance with the local legislation and institutional requirements. The participants provided their written informed consent to participate in this study. Expanded ASC were isolated from 5 patients in which liposuction was performed from the same anatomical area (thigh) for breast reconstruction in disease free conditions. Results included in this work were obtained using cells derived from at least 3 out of the 5 selected patients.

Stromal vascular fraction isolation and ASC expansion were performed as previously published (3). Briefly, a solution of collagenase, 0.15 U/ml final concentration (NB 6 Good Manufacturing Practice grade, SERVA Electrophoresis GmbH, Heidelberg, Germany) was added to the washed lipoaspirate. After 1 hour incubation at 37°C and collagenase neutralization by human albumin solution (Albital 200 g/l, Kedrion S.p.A., Lucca; Italy), the lower phase was recovered and centrifuged at 400 x g for 10 minutes at +4°C. The cell pellet was washed with a solution composed by 10% human albumin, 10% Anticoagulant Acid Citrate Dextrose Solution – A (ACD-A; Haemonetics Corporation, Braintree, MA; USA), 2 U/ml heparin (Epsodilave, HOSPIRA ITALIA S.r.l., Napoli; Italy) in Ringer Lactate solution (S.A.L.F. S.p.A. Laboratorio Farmacologico, Bergamo; Italy). Cell viability was controlled by Trypan blue dye exclusion test and cell identity was confirmed by flow citometry, exactly as previousy published (3). Stromal vascular fraction cells were frozen in autologous or AB serum containing 5% dimethylsulfoxide (CryoSure-DMSO, Li StarFish, MI; Italy). Thawed SVF cells were separately plated in standard T25 tissue culture flasks (BD Biosciences, Bedford, MA; US) with Minimum Essential Medium Eagle - Alpha Modification (α-MEM) (Lonza; Basel, Switzerland) with 100 IU/ml of Penicillin, 100 mg/ml of Streptomycin (both from Merck, Rahway, NJ; US) added with 10% vol/vol FBS or in parallel with 5% vol/vol SRGF. SRGF was used at 5% as we previously demonstrated that further increasing SRGF concentrations failed to confer relevant advantages in terms of ASC proliferation rate (18). Cells were cultured in clinical grade incubators (MCO-19AIC UV; Sanyo, Osaka, Japan) at 37°C, 5% CO_2_ and 100% humidity. Non-adherent stromal vascular fraction cells were removed after 24 hours. Further ASC expansion was performed using 10% FBS or 5% SRGF α-MEM media. In this work, as indicated for specific experiments, SRGF was reduced at concentrations of 0.3; 0.6; 1.25; 2.5% vol/vol. Upon 80–90% confluence, ASC were detached by trypsin-ethylenediaminetetraacetic acid (EDTA) (TrypLe Select 10X, Thermo Fisher Scientific, Waltham, MA; US) and subcultured again. ASC expanded in 5% SRGF and 10% FBS media fully detached from plastic surface, respectively, after 2 and 5 minutes exposure to trypsin-EDTA. Resuspended cells were seeded (at P1 and at each following cell passages) at 2.5–3.0 x 10^3^ cells/cm^2^.

Human hepatocellular carcinoma HepG2 and human fibrosarcoma HT1080 cell lines were expanded in Dulbecco’s modified Eagle’s medium (Lonza) with high glucose and 10% FBS. The human glioblastoma cell line T98G was maintained in Eagle’s minimal essential medium (Lonza) supplemented with Earle’s basic salt solution and 10% FBS. Cells were cultured in clinical grade incubators (MCO-19AIC UV; Sanyo, Osaka, Japan) at 37°C, 5% CO_2_ and 100% humidity. All growth media were supplemented with 100 IU/ml of Penicillin, 100 mg/ml of Streptomycin (both from Merck). Conditioned medium (CM) from each cancer cell line was obtained exposing subconfluent cells to fresh medium containing the appropriate FBS concentration (as indicated in each experimental condition). After 24 hours, the supernatant was collected and centrifuged at 4500xg for 15 minutes. Aliquots were stored at -20°C until use.

### 2.2 Spheroid formation

To expand ASC as spheroids we seeded 2 x 10^4^ ASC/well in low attachment 96 U-bottom well plates (Nunclon Sphera, Thermo Fisher Scientific) in the two different culture conditions, i.e. in presence of 5% SRGF or of 10% FBS. After cell aggregation in spheroids (24 hours), cells were transferred to 35 mm low attachment plates (35 mm Dish Nunclon Sphera, Thermo Fisher Scientific). After additional 24 hours incubation, spheroids were centrifuged for 5 minutes at 450 x g. To dissociate ASC from spheroids, the supernatant was discarded and trypsin-EDTA (1x) was added to spheroids. After 3 minutes incubation (37°C), an excess of complete medium was added to inhibit trypsin activity and cells were centrifuged at 450 x g. ASC expanded in 5% SRGF were dissociated twice with trypsin-EDTA, while 10% FBS were dissociated three times. Finally, the solution was passed through a strainer (40 μm cutoff), centrifuged at 450 x g and washed with PBS. The cell pellet was resuspended in the appropriate medium (i.e. in presence of 5% SRGF or of 10% FBS), and used for the functional assay.

### 2.3 Wound healing assay

ASC (1 x 10^4^ cells) expanded in presence of 5% SRGF or of 10% FBS were seeded in each chamber of a Culture-Insert 2 Well (Ibidi, Gräfelfing; Germany) device, coated with bovine collagen type I (Thermo Fisher Scientific) at the concentration of 50 ng/μl (1 hour incubation at room temperature). After complete adhesion, the separating chamber was removed, allowing spontaneous cell movements toward the empty space. During the assay, α-MEM medium containing 1% FBS or 1% SRGF was applied to reduce as much as possible cell proliferation, without potentially affecting cell motility. After 6 and 24 hours from assay beginning, images were taken by inverted phase contrast microscope (Olympus CKX41, Olympus Italia Srl, Milano; Italy). Thresholded images were analyzed by ImageJ (19) and percent covered area in the middle channel were considered.

### 2.4 Bidimensional chemotaxis migration assay

To assess the impact of a chemotactic stimulus on ASC migration after expansion in presence of 5% SRGF or of 10% FBS, ASC were seeded at low concentration (1.000 total cells) in μ-slide chemotaxis devices (Ibidi). The device is composed of a central narrow channel in which cells are seeded. The channel is flanked on its left and right sides by a wing-shaped reservoir that, in virtue of its geometry, allows the onset of a chemotactic gradient. The central channel was coated with bovine collagen type I (Thermo Fisher Scientific) and after complete adhesion, cells were exposed to oriented gradients of chemotactic stumuli. Serum free α-MEM medium was injected into the left reservoir, while a medium containing only 2.5% FBS, or cancer cell CM containing 2.5% FBS, was introduced into the right reservoir. As technical negative control, in parallel assays, serum free medium (supplemented with antibiotics) was added in both right and left reservoirs. Cell migration was monitored by time course imaging of cells seeded in the middle channel (1 image/30 minutes). Environmental conditions during time lapse imaging were controlled maintaining temperature at 37°C. Manual tracking function of ImageJ was used to define trajectories of moving cells. Datasets of cell coordinates changing over time were analyzed by Chemotaxis and Migration tool (Ibidi). Forward Migration Index (FMI), as calculated by Chemotaxis and Migration tool, was considered as indicator of active cell migration, determined by the chemotactic stimulus. Mean FMI values scored in negative control experiments performed in each condition were closely approximate to 0 (data not shown).

### 2.5 Transwell assay in static conditions

2 x 10^4^ ASC, expanded in presence of 5% SRGF (if not otherwise stated for setup experiments) or of 10% FBS were seeded in duplicate in transwell chambers (Transwell Permeable Support; Costar, WA, US), previously coated with bovine collagen type I (Thermo Fisher Scientific) at the concentration of 50 ng/μl (1 hour incubation at room temperature). Transwell chambers were equipped with polyester (PET) or polycarbonate (POLY) septa (both 8.0 micron cutoff pore size). To trigger cell migration, complete medium containing 5% FBS (if not otherwise stated) or cancer cell CM (with the same FBS concentration) were used as chemoattractants. Serum free medium was applied to the upper compartment. After 24 hours in cell culture incubators (Sanyo) at 37°C, 5% CO_2_ and 100% humidity, migration was stopped fixing cells with 4% cold (+4°C) paraformaldehyde (Sigma-Merk, St Louis, MO; US). ASC on both septum sides were stained with 4′,6-diamidino-2-phenylindole (DAPI) (Thermo Fisher Scientific) and visualized by a fluorescence microscope (Nikon, Tokyo, Japan) equipped 10x objective and with camera for image capture. Images, taken in the middle of the septum almost covered the whole area of migration septa. Pictures were then analyzed by Imagej and total ASC (nuclei of migrated and non-migrated cells) were counted by the “Analyze particles” plugin. Objects size threshold was set over 50 squared pixels to avoid inclusion of artifact dots in the final count. Thereafter, non-migrating ASC were scraped from the upper part of the septum by a cotton swab, and additional images of migrated cells were captured and analyzed as above. Transmigration index was estimated as percent ratio between migrated cells (post scrape) and total cells (before scrape) in each chamber.

### 2.6 Transwell assay in dynamic millifluidic conditions

To assess ASC migration potential in millifluidic conditions, we took advantage of the LifeBox2 device (Ivitech, LU, Italy), equipped with the peristaltic pump LiveFlow (Ivitech), compatible with the incubator environment. The LiveBox2 chamber is composed of 2 compartments, separated by a collagen coated (see above) PET septum. Resembling the architecture of static transwell chambers, the lower compartment was filled with chemoattractant media composed of complete medium added with 10% FBS or of cancer cell CM (with the same FBS concentration). Flow was not applied to the lower compartment closing tightly together inlet and outlet catheters. Otherwise, serum free medium flow (500 μl/minute, maximal flow rate) was applied to the upper compartment and ASC, previously expanded in presence of 5% SRGF or of 10% FBS, were injected into the close circuit through the reservoir. The final concentration of circulating cells was 2.4 x 10^4^ ASC/ml. After 24 hours, the LiveBox2 chamber was disassembled taking advantage of the appropriate device to avoid disruption or perturbation of the adherent cell layer. The PET septum was carefully removed and cells were fixed by 4% cold (+4°C) paraformaldehyde (Sigma-Merk), stained with DAPI (Thermo Fisher Scientific) and analyzed as abovementioned for static assays. In order to analyze the major part of migration surface area, five pictures were taken in each septum before and after non-migrating cell removal by cotton swab. ASC transmigration index (%) was calculated by the same formula applied for static transwell assays.

### 2.7 Transient transfection

ASC were seeded in transwell chambers and a DNA vector (pRP-CMV-hACTA2; from Vectorbuilder; Chicago, IL, US), encoding for aSMA in fusion with the reporter green fluorescent protein (aSMA-GFP), was transfected using Lipofectamine Stem Transfection Reagent (Thermo Fisher Scientific) and following manufacturer’s instructions. Lipofectamine was chosen as this reagent couples adequate transfection efficiency and reduced impact on cell viability. As control condition, cells were transfected with an empty vector (pRP-CMV-GFP; from Vectorbuilder) encoding for the only green fluorescent protein (GFP). A ratio of 1μg DNA to 2μl Lipofectamine was chosen to perform the transfection. To setup the appropriate protocol, 15 or 30 ng DNA/seeded cell were administered. Protein expression, was evaluated by western blot or immunofluorescence assays, 24 hours after transfection.

### 2.8 Western blot assay

Proteins were extracted from cells using a lysis buffer, composed by PBS + 0.5% NP40 non ionic detergent (Thermo Fisher Scientific) supplemented with protease and phosphatase inhibitors (Merck KGaA). Protein concentrations were measured using a BCA protein assay kit (Thermo Fisher Scientific) and a proper amount of cell lysate was loaded into a 4–20% protean TGX gel (Bio−Rad, Hercules, CA, USA). After electrophoresis, proteins were transferred to 0.2 μm PVDF membrane using a Trans−Blot Turbo device (BioRad, Hercules, CA, USA). Membranes were blocked for 1 hour with 5% milk proteins (Blotting Grade Blocker −BioRad, Hercules, USA) in Tris-buffered saline with 0.1% Tween 20 detergent (TBST). Primary antibodies were added at appropriate concentration in 5% milk in TBST overnight at 4°C. The following antibodies were used: anti FAK (1:1000, Abcam, Cambridge, UK, #ab76496); anti phospho FAK Y397 (1:1000, Abcam, #ab81298); anti αSMA (1:1000, Sigma-Merk, #A5228); anti VINC (1:3000, Abcam, #ab129002); Anti PAX (1:1000, Abcam, #ab32084); anti−FLNA (1:1000, Proteintech, Manchester, UK, #67133−Ig), anti−Phosphorylated FLNA S2152 (1:1000, ABclonal, Woburn, MA, USA #AP0783), and anti− glyceraldehyde-3-phosphate dehydrogenase (GAPDH) (1:3000, Merkmillipore, Burlington, NJ, USA, #CB1001); Anti GFP (1:1000, Roche, Basel, Switzerland, #11814460001). After the incubation with proper secondary antibodies anti−mouse HRP (1:1000, GE Healthcare, Milan, Italy, #GENA931) or anti−rabbit HRP (1:1000, SouthernBiotech, Birmingham, AL, USA, #4030−05), the immunoreactive bands were visualized with Amersham ECL Prime Western Blotting Detection Reagent (Cytiva, Marlborough, MA, USA) and Chemidoc Imaging System (BioRad, Hercules, CA, US). Relative band quantification was performed by the ImageJ software.

### 2.9 Immunofluorescence assays

ASC were seeded on ethanol treated cover glasses and transfected as above mentioned. After 24 hours, cells were fixed with 4% cold paraformaldehyde (Sigma-Merk), permeabilized by 0.1% Triton X100 (Sigma-Merck), blocked with 2% BSA and incubated with Anti αSMA (1:250, Sigma-Merk, #A5228) and with Rhodamine Red™-X (RRX) AffiniPure™ F(ab’)_2_ Fragment Goat Anti-Mouse IgG (H+L) (1:300, Jackson Immunoresearch, PA, US) as secondary antibody. As control condition, fixed and permeabilized cells were stained with the only secondary antibody. After nuclei counterstaining by DAPI (Thermo Fisher Scientific), glass coverslides were mounted by ProLong Antifade mounting medium (Thermo Fisher Scientific). Cells were analyzed by fluorescent microscopy (Nikon).

### 2.10 Statistical analysis

In this work data were reported as mean ± S.E.M. Statistical differences between two mean values were analyzed by Student’s T-test, as detailed in each figure legend and in results. Statistical mean differences between three or more groups were analyzed by ANOVA test, as detailed in each figure legend and in results. When appropriate, *post hoc* analysis was performed by Tukey’s Honest Significant Difference (HSD) test. Correlation between two variables was analyzed by linear regression analysis.

## 3 Results

### 3.1 Enhanced chemoattractant-mediated planar migration of SRGF-ASC

**Figure 1:**
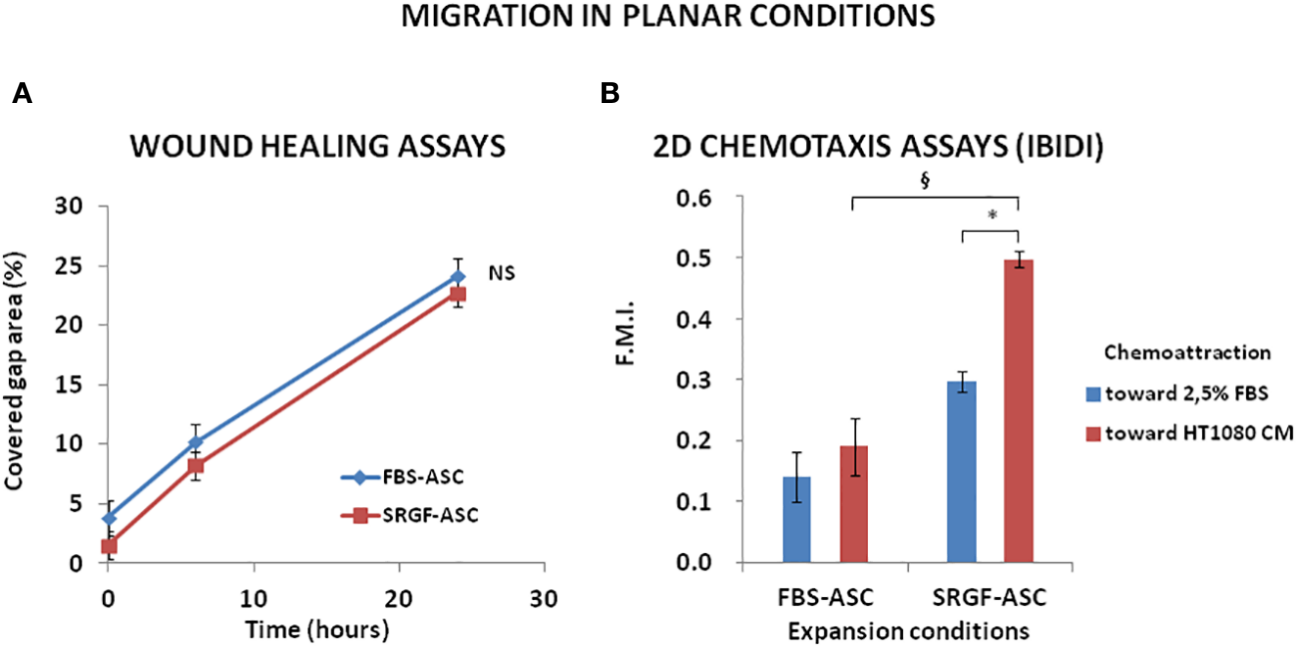
FBS-ASC and SRGF-ASC migration in planar conditions, with or without chemotactic stimulus. **(A)** shows, in a time course analysis, that, without chemotactic stimulus, there is not significant difference between FBS-ASC and SRGF-ASC motility rate. (Student’s T-test for paired data between 6 and 24 hours migration mean results). NS, not significant. **(B)** shows FBS-ASC and SRGF-ASC actively migrated toward 2.5% FBS and toward the HT1080 CM (F.M.I., mean Forward Migration Index). SRGF-ASC, but not FBS-ASC, showed greater migration potential toward HT1080 CM, *vs* 2.5% FBS (p=0.008; Two-Way Factorial ANOVA for Independent Samples; *, p=0.03 *post hoc* analysis by Tukey’s HSD test). Independently from chemoattraction type, SRGF-ASC showed a far greater migration capacity *vs* FBS-ASC (^§^, p=0.02, *post hoc* analysis by Tukey’s HSD test).

In this work, we investigated the impact of the medium additive SRGF on migration potential of ASC. Thus, we expanded ASC in presence of 5% SRGF (SRGF-ASC) and, as control condition, in presence of 10% FBS (FBS-ASC). To analyze SRGF impact on ASC motility, in planar conditions and in absence of a chemotactic stimulus, we performed standard wound healing assays. **Figure 1A** shows a time course analysis of covered area changes in the artificial gap between cells: no significant differences in motility rate were shown between FBS-ASC and SRGF-ASC (Student’s T-test for paired data between 6 and 24 hours migration mean results). Afterward, still in planar configuration, we exposed cells to a chemotactic gradient created by HT1080 CM and, as reference, by a fresh cell culture medium containing 2.5% FBS. The 2.5% FBS concentration was selected as ASC migration potential was sub-maximally stimulated in presence of 5% FBS as chemoattractant (data not shown). As displayed in **Figure 1B**, both FBS-ASC and SRGF-ASC actively migrated toward 2.5% FBS and toward HT1080 CM. Noteworthy, SRGF-ASC, but not FBS-ASC, showed significantly greater forward migration index toward HT1080 CM, when compared to the reference chemoattraction medium containing 2.5% FBS (p=0.008 Two-Way Factorial ANOVA for Independent Samples; *, p=0.03, *post hoc* analysis by Tukey’s HSD test). Moreover, SRGF-ASC migration capacity toward HT1080 CM was significantly (^§^, p=0.02, *post hoc* analysis by Tukey’s HSD test) higher when compared to FBS-ASC. In contrast, no differences in ASC forward migration index were identified when experiments were performed using complete cell culture medium, or HT1080 CM, both containing 10% FBS (data not shown).

### 3.2 Improved transmigration potential of SRGF-ASC: relationships with chemoattractant concentrations

**Figure 2:**
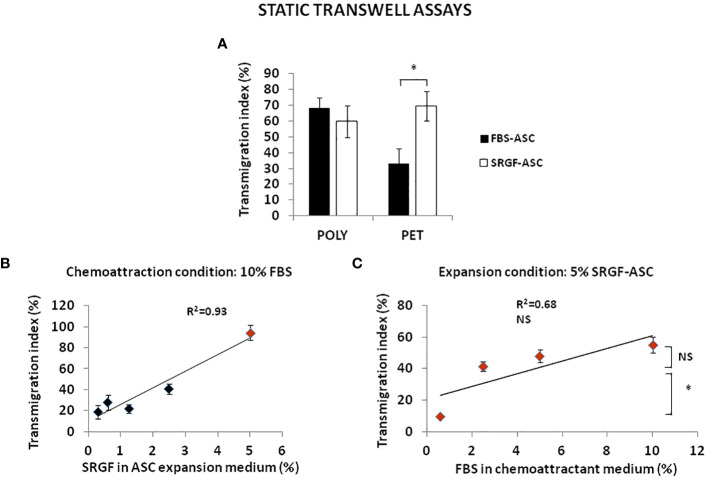
FBS-ASC and SRGF-ASC transmigration potential, analyzed by standard transwell assay in static conditions. **(A)** shows that both FBS-ASC and SRGF-ASC strongly migrated across polycarbonate (POLY) septa, without significant differences. When polyester (PET) septa were used, SRGF-ASC transmigration potential was markedly and significantly higher *vs* FBS-ASC. (p=0.04; Two-Way Factorial ANOVA for Independent Samples; *, p=0.03 *post hoc* analysis by Tukey’s HSD test). **(B)** shows the linear relationship between SRGF concentrations in ASC expansion medium (5 days culture) and ASC transmigration potential, using 10% FBS as chemoattractant (Linear regression analysis: p=0.02; R^2 ^= 0.93). **(C)** demonstrates that FBS concentrations in the chemoattraction medium are not directly correlated to transmigration potential of 5% SRGF-ASC (Linear regression analysis: not significant; R^2 ^= 0.68). Differences between mean transmigration indexes measured when ASC migrated toward 2.5% FBS or 10% FBS were not significant. Otherwise, cell migration potential was significantly decreased when 0.6% FBS was used. (p=0.03 ANOVA for repeated measures; *, p=0.04 *post hoc* analysis by Tukey’s HSD test). NS, not significant.

We analyzed transmigration potential of expanded ASC by standard transwell assay. To setup the experimental conditions, we tested the impact on ASC migration taking advantage of different septa separating upper and lower transwell chamber. In such experiments, we used as chemoattractant a cell culture medium containing 10% FBS. As displayed in **Figure 2A**, both FBS-ASC and SRGF-ASC showed a comparable very high migration potential across POLY septa. On the other hand, when PET septa were used, SRGF-ASC transmigration potential toward the chemoattractive stimulus was markedly and significantly higher than FBS-ASC (p=0.04; Two-Way Factorial ANOVA for Independent Samples; p=0.03 *post hoc* analysis by Tukey’s HSD test). For such reasons, we further investigated the impact of SRGF on ASC transmigration potential using PET septa only. We cultured, for at least 5 days, our ASC in cell culture media containing different concentrations (0.3; 0.6; 1.25; 2.5; 5% vol/vol) of SRGF as medium additive. As depicted in **Figure 2B**, using the same chemoattractant stimulus, i.e. a cell culture medium containing 10% FBS, migration potential of SRGF-ASC was shown to be significantly correlated to percent SRGF concentrations used for previous ASC expansion (Linear regression analysis: p=0.02; R^2 ^= 0.93). Analogous assays involving ASC expanded in presence of FBS were not feasible as decreasing FBS concentrations below 10% caused an unacceptable reduction of cell proliferation rate and/or viability (data not shown). Otherwise (**Figure 2C**), focusing on ASC expanded in presence of 5% SRGF, the FBS concentration in the chemoattraction medium (lower chamber), was not directly correlated to ASC migration potential (Linear regression analysis: not significant; R^2 ^= 0.68). In particular, a steep decrease in cell migration potential was observed when FBS was used as chemoattractant at concentrations below 2.5% (p=0.03 ANOVA for repeated measures; *p=0.04 *post hoc* analysis by Tukey’s HSD test).

### 3.3 Increased transmigration of SRGF-ASC toward cancer derived chemoattraction: results in static and dynamic conditions

**Figure 3:**
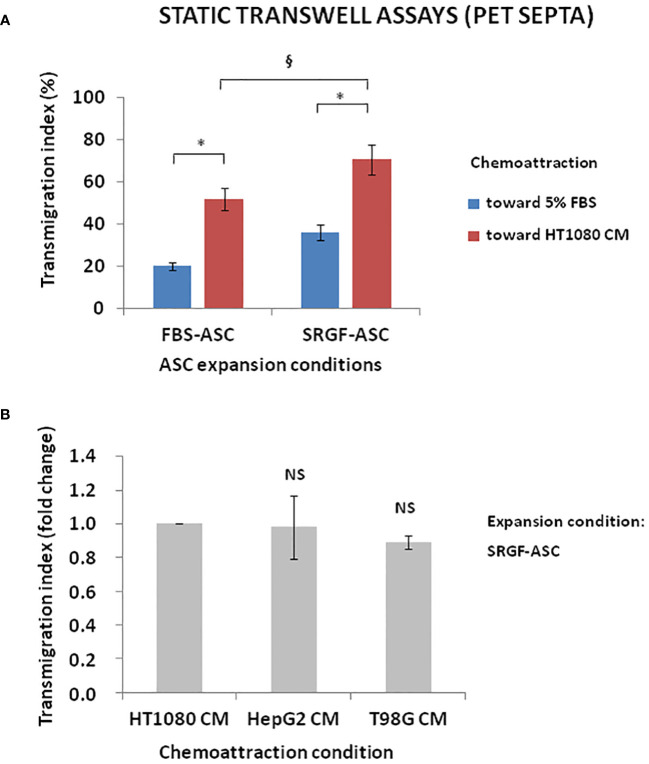
ASC transmigration in static conditions toward cancer cell CM. **(A)** shows that both FBS-ASC and SRGF-ASC were characterized by a greater migration potential toward HT1080 CM than toward 5% FBS (p=0.002; Two-Way Factorial ANOVA for Independent Samples; *, p=0.04 *post hoc* analysis by Tukey’s HSD test). SRGF-ASC better migrated toward HT1080 CM when compared to FBS-ASC (^§^, p=0.03 *post hoc* analysis by Tukey’s HSD test). **(B)** shows that SRGF-ASC migration potential (fold change) toward T98G and HepG2 CM was not different, when compared to HT1080 (NS, ANOVA for independent measures). NS, not significant.

**Figure 4:**
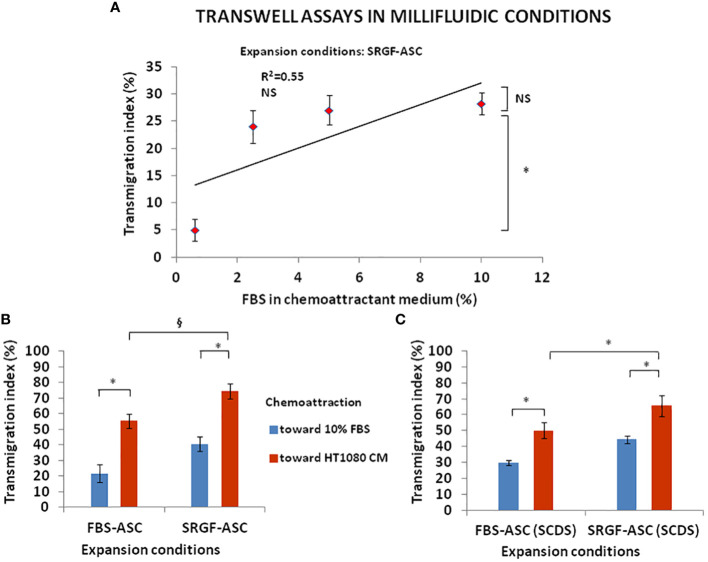
FBS-ASC and SRGF-ASC migration potential in millifluidic conditions. **(A)** shows that, in millifluidic conditions, migration rate of SRGF-ASC was not directly related to FBS chemoattractant concentrations (Linear regression analysis: not significant; R^2 = ^0.55), and that migrating cells markedly decreased when FBS was reduced below 2.5% (p=0.008 ANOVA for repeated measures; *p=0.006 *post hoc* analysis by Tukey’s HSD test). NS, not significant. **(B)**, shows that both FBS-ASC and SRGF-ASC better migrated toward HT1080 CM, *vs* the reference chemoattraction medium (p=0.008 Two-Way Factorial ANOVA for Independent Samples; *, p=0.04 *post hoc* analysis by Tukey’s HSD test). The migration potential of SRGF-ASC toward HT1080 CM was significantly higher, *vs* FBS-ASC (^§^, p=0.03 *post hoc* analysis by Tukey’s HSD test). **(C)** shows transmigration potential, in millifluidic tranwell system, of FBS-ASC and SRGF-ASC expanded as single cells dissociated from spheroids (SCDS). SRGF-ASC better migrated than FBS-ASC, both toward 10% FBS or toward HT1080 CM. HT1080 CM increased migration potential of both FBS-ASC and SRGF-ASC *vs* the reference chemoattraction condition (p=0.01 Two-Way Factorial ANOVA for Independent Samples; *, p=0.04 *post hoc* analysis by Tukey’s HSD test). The migration capacity of SRGF-ASC toward HT-1080 medium was significantly higher, when compared to FBS-ASC (*, p=0.04 *post hoc* analysis by Tukey’s HSD test).

We investigated the impact of cancer cell derived chemotactic stimulus on transmigration of SRGF-ASC. Results summarized in **Figure 3A** were obtained using standard static transwell assays. Both FBS-ASC and SRGF-ASC showed a greater migration potential toward HT1080 CM, than in the direction of a reference medium that contained only 5% FBS (p=0.002; Two-Way Factorial ANOVA for Independent Samples; *, p=0.04 *post hoc* analysis by Tukey’s HSD test). Interestingly, SRGF-ASC better migrated toward HT1080 CM, when compared to FBS-ASC (^§^, p=0.03 *post hoc* analysis by Tukey’s HSD test). In turn, we analyzed SRGF-ASC migration potential toward T98G and HepG2 CM: as shown in **Figure 3B**, when compared to HT1080, SRGF-ASC equally migrated toward HepG2 and T98G derived chemotactic stimuli (NS, not significant, ANOVA for independent measures). Thus, in order to better define SRGF impact on ASC diapedesis potential, we took advantage of a challenging migration assay, as the millifluidic transwell chamber. In such experimental setting, the bottom chamber (i.e. below the PET septum) containing the chemotactic medium, was kept in static conditions. Otherwise, expanded ASC were allowed to flow through the upper chamber within a close circuit system, perfused by a peristaltic pump. As displayed in **Figure 4A**, we demonstrated that, in analogy with abovementioned results, migration rate of SRGF-ASC was not directly related to FBS concentrations in the lower chamber (Linear regression analysis: not significant; R^2 ^= 0.55) and that the fraction of migrating cells markedly decreased when FBS was reduced below 2.5% (p=0.008 ANOVA for repeated measures; *p=0.006 *post hoc* analysis by Tukey’s HSD test). Millifluidic experimental conditions were considered as most challenging for ASC adhesion and migration and, thus, CM and reference medium containing 10% FBS were adopted in the experimental setup. As reported in **Figure 4B**, both FBS- and SRGF-ASC displayed an higher migration potential, through the porous septum, toward HT1080 CM, when compared to the reference chemoattraction medium (p=0.008 Two-Way Factorial ANOVA for Independent Samples; *, p=0.04 *post hoc* analysis by Tukey’s HSD test). Moreover, the migration potential of SRGF-ASC toward HT1080 CM was significantly higher, when compared to FBS-ASC (^§^, p=0.03 *post hoc* analysis by Tukey’s HSD test). To further complete the analysis of SRGF impact on ASC migration potential, FBS- and SRGF-ASC were expanded as floating spheroids, to be in turn dissociated into single cells (SCDS) and injected in the millifluidic tranwell system. Also in such conditions (**Figure 4C**), HT1080 CM increased migration potential of both FBS- and SRGF-ASC, when compared to the reference chemoattraction condition (p=0.01 Two-Way Factorial ANOVA for Independent Samples; *, p=0.04 *post hoc* analysis by Tukey’s HSD test). Again, the migration capacity of SRGF-ASC toward HT-1080 medium was significantly higher, when compared to FBS-ASC (*, p=0.04 *post hoc* analysis by Tukey’s HSD test).

### 3.4 Increased migration capacity of SRGF-ASC is mediated by αSMA downregulation

**Figure 5:**
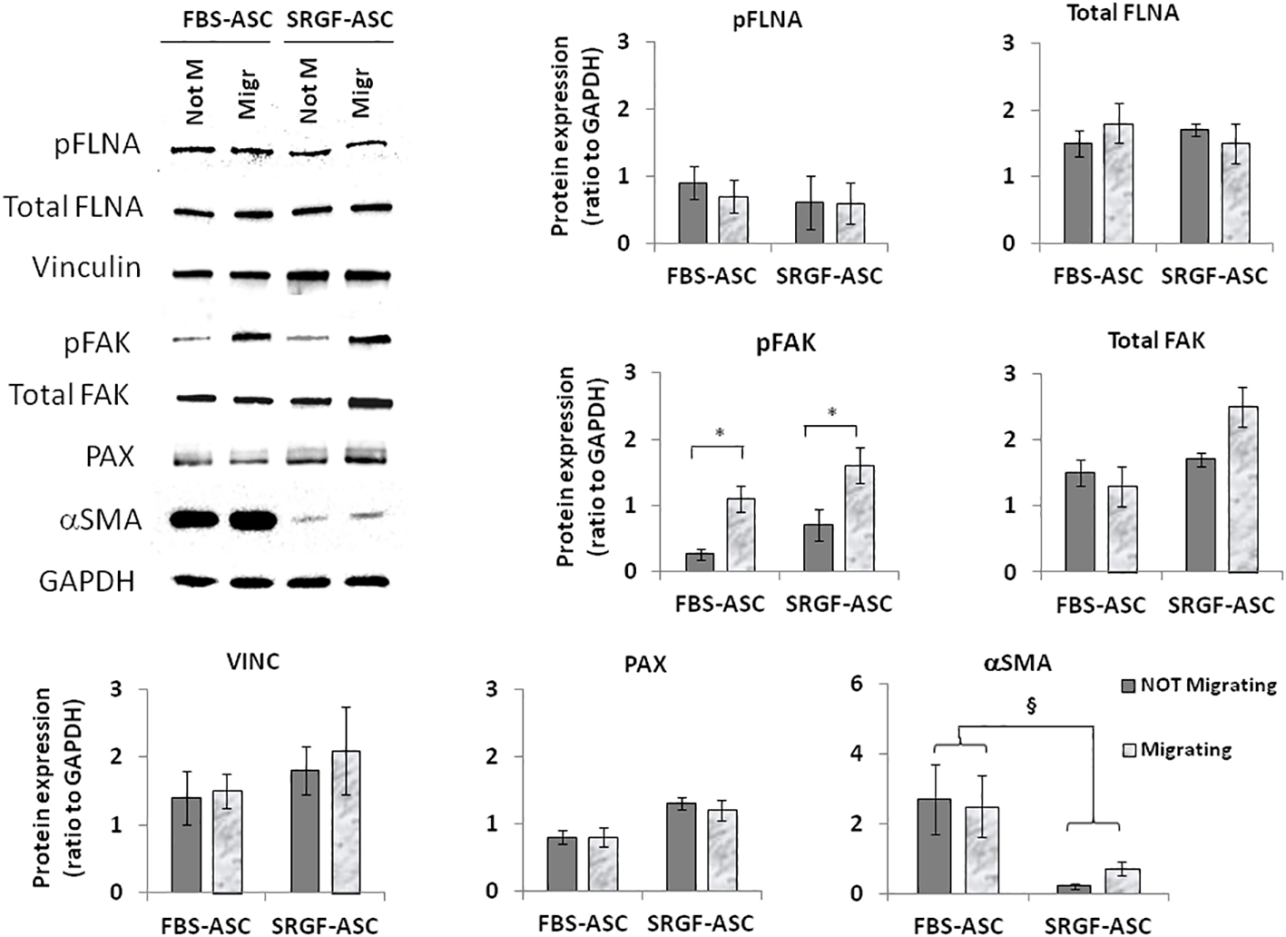
Representative images and quantification of western blot analysis performed on not-migrating (Not M) or migrating (Migr) FBS-ASC and SRGF-ASC. Migration was triggered using a 10% FBS medium. The chemotactic stimulus increased the availability of phosphorylated FAK (p=0.03 Two-Way Factorial ANOVA for Independent Samples; *, p=0.03 *post hoc* analysis by Tukey’s HSD test), while total FAK expression levels were not differently regulated by chemoattraction, or expansion conditions. Expression levels of VINC, phosphorylated FLNA and PAX were not affected by chemotaxis or by ASC expansion conditions. Otherwise, in FBS-ASC, αSMA availability was extremely higher than in SRGF-ASC, independently from ASC exposure to a chemotactic stimulus (p=0.01 Two-Way Factorial ANOVA for Independent Samples; ^§^, p=0.005 *post hoc* analysis by Tukey’s HSD test).

**Figure 6:**
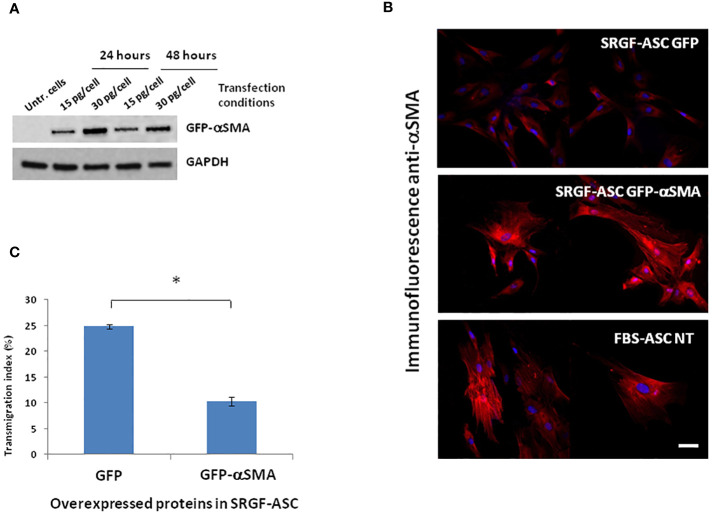
Morphology and migration potential of ASC overexpressing GFP-αSMA. **(A)** reports representative western blot showing that optimal production of GFP-αSMA was achieved 24 hours after transfecting 30 ng DNA per cell. **(B)** shows representative images of immunofluorescence assays perfomed using an anti αSMA primary antibody on fixed cells. Images of control cells stained with the only secondary rhodamin labeled antibody showed no signal (not reported). Top panel: SRGF-ASC transfected by a GFP encoding vector (control). Middle panel: SRGF-ASC overexpressing GFP-αSMA. Lower panel: non-transfected (NT) FBS-ASC. After GFP-αSMA transfection, SRGF-ASC cells recapitulated FBS-ASC morphology and expressed a markedly higher amount of intracellular αSMA, when compared to GFP transfected SRGF-ASC. **(C)** shows that migration capacity of SRGF-ASC overexpressing GFP-aSMA was strongly reduced *vs* SRGF-ASC overexpressing the only GFP protein. Only GFP or GFP-aSMA expressing cells were imaged by fluorescence microscopy and considered for transmigration index calculation (*, p=0.009; Student’s T-test for paired data). White solid bar, 10 μm.

We attempted to elucidate potential mechanisms underlying the impact of SRGF on migration capacity of ASC. We analyzed, by western blot, expression level of selected proteins extracted from FBS- and SRGF-ASC cultured in transwell chambers, with or without a chemotactic stimulus (10% FBS medium). As expected, the chemotactic stimulus significantly increased (**Figure 5**), the availability of phosphorylated FAK (p=0.03 Two-Way Factorial ANOVA for Independent Samples; *, p=0.03 *post hoc* analysis by Tukey’s HSD test), while total FAK expression levels were not differently regulated by chemoattraction, or expansion conditions. Expression levels of VINC, phosphorylated FLNA, total FLNA, and PAX were not affected by the chemotaxis, and neither by ASC expansion conditions (**Figure 5**). Otherwise, in SRGF-ASC, αSMA availability was extremely lower than in FBS-ASC, independently from ASC exposure to a chemotactic stimulus (**Figure 5**) (p=0.01 Two-Way Factorial ANOVA for Independent Samples; ^§^, p=0.005 *post hoc* analysis by Tukey’s HSD test). To challenge the hypothesis that SRGF increases ASC reducing αSMA levels, we overexpressed GFP-αSMA in our SRGF-ASC. As shown in **Figure 6A**, optimal production of GFP-αSMA was achieved 24 hours after transfection of 30 pg of DNA vector per seeded cell. As demonstrated by immunofluorescence assay (**Figure 6B**), after GFP-αSMA transfection, SRGF-ASC cells expressed a markedly higher amount of intracellular αSMA, when compared to non transfected cells. Noteworthy, SRGF-ASC overexpressing GFP-αSMA recapitulated cell morphology of FBS-ASC (**Figure 6B**). Thus, 24 hours after DNA delivery to SRGF-ASC, we initiated a transwell assay lasting 24 additional hours. As demonstrated in **Figure 6C**, migration capacity of SRGF-ASC overexpressing GFP-αSMA was strongly reduced when compared to SRGF-ASC expressing the only GFP protein (p=0.009; Student’s T-test for paired data).

## 4 Discussion

ASC can be used for cell therapy applications in several clinical settings in virtue or their homing potential toward inflammation sites and cancer masses (2). In fact, they can be potentially used as drug delivery vehicles (20) or as anti-inflammatory agents specifically favouring local tissue regeneration and microenvironment repair (21). Migration is one of the basic steps of the ASC homing process toward the target (22). Expansion of ASC is required to apply such cell product in clinical settings and it must be performed in compliance with GMP guidelines, preserving integrity of ASC biological features. SRGF was previously shown as a suitable ancillary product to expand ASC for potential applications in human cell therapy (3). Such growth factor mixture, in fact, is devoid of animal components and it can be considered as a standardized ancillary product, suitable for the GMP compliant and consistent expansion of ASC (5). SRGF can strongly increase ASC proliferation rate (3), but the impact of such compound on ASC migration was not previously assessed. Thus, this work was principally aimed to investigate the impact of SRGF on motility and migration properties of ASC after *ex vivo* expansion in GMP compatible conditions. We adopted 5% (vol/vol), as highest SRGF concentration in cell culture, as we previously showed (3) that further increasing the concentration of such ancillary product didn’t improve biological features of such cells. We here showed that, in absence of a chemotactic stimulus, SRGF failed to significantly affect cell motility, i.e. the rate of gap closure in wound healing assays. Previous publications investigated the impact of medium supplementation with an ancillary product defined as platelet lysate. This product shares similarities with SRGF and, in accordance with our results, authors (23) showed that the rate of wound healing was not significantly modified by platelet lysate addition to the cell culture medium. Nevertheless, in scratch assays performed with mesenchymal stem cells derived from rodents (24), platelet lysate supplementation to the cell culture medium was shown to improve gap closure rate. Interestingly, our results showed that ASC expansion in a 5% SRGF containing medium, improved cell migration properties toward a chemotactic gradient, both in planar 2D conditions, or in standard transwell assays. In the planar 2D setting, ASC migration potential was sub-maximally stimulated by 5% FBS as chemoattractant (data not shown), and this flattened differences between FBS- and SRGF-ASC. After preliminary assays, we empirically selected 2.5% FBS as chemoattractant concentration in planar 2D chemotaxis assay: in such conditions, differences between FBS- and SRGF-ASC were clearly evident. Interestingly, we found that SRGF-ASC were particularly attracted by different growth media in which previously expanded cancer cell lines released chemoattractive substances. In this simplified model, such condition resembles the process of ASC tumor specific homing. At our knowledge this is the first report directly showing that ASC expansion in presence of human growth factors derived from platelets can amplify cell migration capacity toward an artificial chemotactic stimulus or toward cancer cells. A previous work (25) showed that, after expansion in presence of platelet lysate, MSC derived from Wharton’s jelly better migrated toward primary untransformed cells as fibroblasts. Only indirect evidence derived from microarray analysis previously showed that platelet lysate can downregulate transcription of migration related genes, when compared to FBS expanded ASC (26). Otherwise, in a previous work, only a statistical prediction model, considering cell dimension, proliferation rate and contact inhibition, suggested that ASC seeded at low density in presence of platelet lysate could display enhanced migration capacity (27). Otherwise, previous papers focused on platelet lysate as source of chemoattractive stimuli. In a recent work (28), MSC derived from humbilical cord and expanded in a medium containing 10% FBS better migrated, toward media containing platelet lysate at higher concentrations. The same work (28) also demonstrates that migration related genes were significantly upregulated. Similarly, migration of rat MSC, expanded in 10% FBS, was significantly stimulated by platelet lysate, as chemoattractant (29). Interestingly, our SRGF expanded ASC were equally and strongly affected by the chemoattraction exerted by each selected cancer cell line, as HT-1080 but also T98G and HepG2. In a previous work (7), we showed that, in microfluidic conditions, direct adhesion of SRGF-ASC on a monolayer of hepatocarcinoma cells was far weaker than on adherent HT-1080 and T98G. Such results are not in contrast with the present work as, we here investigated chemotaxis, but not direct cell-cell interaction, as ASC homing mechanism. Nevertheless, further investigations are required to better characterize the capacity of different hepatocarcinoma cell lines to attract ASC. As peculiar feature of this work, we also explored migration potential of our SRGF-ASC taking advantage of an *in vitro* millifluidic system, closely recapitulating microenvironmental and reologic conditions occurring *in vivo*. This system can evaluate migration properties considering also docking and firm adhesion of flowing cells onto the porous substrate. Even in this challenging conditions, SRGF-ASC demonstrated to be more prone to diapedesis than FBS-ASC, especially toward cancer cells. In these experiments FBS chemoattractant was set at 10% FBS, while in static experiments FBS was maintained at 5%. Noteworthy, we demonstrated that, both in static and dynamic settings, ASC migration is affected only when FBS chemoattractant concentration is below 5%. To perform such assays in millifluidic conditions, we chose the higher concentration (10%) of chemoattractant FBS, considering the biological complexity of the assay itself. Expanding ASC as floating spheroids in static conditions can improve anti-inflammatory, angiogenic, and tissue reparative/regenerative effects of mesenchymal stem cells (30), in turn potentially improving cell stemness. We aimed to assess if SRGF can improve ASC migration properties also when cells were expanded as spheroids, and we directly assessed this issue in the most challenging conditions of millifluidic transwell assays. We showed that, in comparison with ASC expanded as adherent single cells, ASC expansion in the condition of floating spheroid didn’t confer additional advantage in terms of migration potential. Nevertheless, we confirmed that ASC derived from spheroids expanded in presence of SRGF were characterized by improved migration properties when compared to FBS expanded counterparts, especially toward cancer cells.

In order to elucidate a potential mechanism underlying the increased migration capacity of SRGF-ASC in presence of a chemoattractive gradient, we compared expression of selected candidate proteins involved in ASC migration machinery. We evidenced the upregulation of FAK phosphorylation after cell exposure to the migration stimulus. This effect was paralleled by only a minimal increase of total FAK content in migrating SRGF-ASC. Several previous publications demonstrated that cell exposure to a chemoattraction stimulus can induce FAK phosphorylation (31, 32): thus, the observed effect was considered as a demonstration that ASC actually responded to the applied chemotactic gradient. In the analysis of molecular mechanisms potentially explaining SRGF mediated impact on ASC migration capacity, ASC were exposed to the only chemotactic stimulus determined by 10% FBS, i.e. not by cancer cell CM. Such choice was taken to simplify our model, considering the evidence that CM just increased migration properties of our SRGF cells, without differential effects on such cellular feature. In our results, ASC expansion in presence of SRGF failed to deregulate VINC and PAX expression. As above mentioned, expression of these two proteins is mandatory for cell migration, but they both appear to be not involved in the SRGF mediated increase of ASC migration potential. We previously showed that levels of phosphorylated FLNA are increased in highly migrating cells, as MSC derived from multiple myeloma patients (17). In present results, phoshorilated FLNA levels were not affected by SRGF or FBS addition to the cell culture medium or by the migration stimulus. Such evidence can be explained considering the different source of stem cells we here analyzed: multiple myeloma cells are, in fact, known to establish a cross-talk with surrounding MSC, in the bone marrow, that confers peculiar migration and proliferative properties to such stem cells (33, 34). Otherwise, our ASC were isolated form stromal vascular fraction derived from adipose tissue that was harvested from the thigh, i.e. distally form neoplastic lesions. Moreover, at the moment of liposuction, enrolled breast cancer patients were in a disease-free condition. Otherwise, independently from application of the chemotactic stimulus, αSMA expression levels were barely detectable in SRGF-ASC, while in FBS-ASC its expression was markedly higher. A previously published paper (35) confirms that MSC, expanded in presence of platelet derived growth factors, can be characterized by diminished occurrence of stress fibers, composed of actin filaments. Thus, we challenged the hypothesis that reduced αSMA expression could improve ASC migration. Artificial silencing of αSMA expression in FBS-ASC would not be technically feasible as transient transfection of such cells is poorly efficient (36). So, we overexpressed αSMA in our SRGF-ASC, and we showed that cells rapidly changed their morphology and stress fiber composition, resembling features of FBS-ASC. More importantly, we showed that migration capacity was impaired in SRGF-ASC cells transiently overexpressing αSMA: this strongly suggests that changes in αSMA availability could mediate the increase of migration potential demonstrated in SRGF-ASC. In the cell model of smooth muscle cells, selective upregulation of αSMA decreased cell proliferation and migration through Rac1 inhibition, even though stress fiber formation was not affected (16). An additional work showed that (37) αSMA inhibition in fibroblasts could increase migration rate of such cells, altering the architecture of focal adhesions and, thus, possibly immobilizing cells on the adhesion surface. Other works showed that migrating liver cells exhibited more intense expression of αSMA (38), thus suggesting that such protein is required for appropriate cell contractility. We may speculate that SRGF modulates αSMA synthesis at appropriate levels, correctly mediating cell adhesion, stiffness and contractility properties, in turn allowing efficient cell migration. This work has not fully elucidated the impact of SRGF on ASC motility and migration and especially on molecular mechanisms involved in this scenario. The panel of screened proteins involved in the migration machinery was limited and we can’t exclude the contribution of other factors in the SRGF mediated regulation of ASC migration potential. Moreover, further evidences in animal models could additionally shed light on SRGF mediated effects on expanded ASC, better characterizing the capacity of such cells to reach target cancer and/or inflammation sites. Results enclosed in this work can only show that, expanding ASC in presence of SRGF, could provide a cell therapy product, compatible with GMP guidelines, characterized by improved cell migration capacity and, thus, by potential utility in cancer therapy. Additional steps are required to suggest a potential clinical application of these evidences. Mesenchymal stem cells are known to play a dual role on tumors (39), nevertheless. the influence of SRGF-ASC on target cancer cells was not investigated by the present study. Similarly, results enclosed in this work didn’t assess whether SRGF-ASC can play a beneficial and appropriate function on immune system effector cells (40). Further experimental campaigns are required to confirm our observations and their potential implications on cancer and inflammatory diseases: nevertheless, we previously showed that SRGF expanded ASC can be genetically modified with elevate efficiency by the electroporation approach (36). Thus, we can conclude that appropriately engineered SRGF-ASC could potentially represent a suitable tool for targeted drug delivery against cancer growth and for the control of inflammatory diseases with reduced systemic impact on off-target sites.

## Data availability statement

The raw data supporting the conclusions of this article will be made available by the authors, without undue reservation.

## Ethics statement

The studies involving humans were approved by Ethics Committee of CRO Aviano, National Cancer Institute, IRCCS. The studies were conducted in accordance with the local legislation and institutional requirements. The participants provided their written informed consent to participate in this study.

## Author contributions

FA: Conceptualization, Data curation, Funding acquisition, Investigation, Methodology, Visualization, Writing – original draft. CV: Conceptualization, Data curation, Investigation, Methodology, Validation, Writing – original draft. EL: Formal analysis, Visualization, Writing – review & editing. FR: Formal analysis, Validation, Writing – review & editing. MiM: Formal analysis, Validation, Writing – review & editing. SM: Formal analysis, Resources, Writing – review & editing. MaM: Funding acquisition, Project administration, Writing – review & editing. CD: Funding acquisition, Project administration, Resources, Supervision, Validation, Writing – review & editing.
